# Abnormal Vestibulo–Ocular Reflex Function Correlates with Balance and Gait Impairment in People with Multiple Sclerosis

**DOI:** 10.3390/audiolres14050067

**Published:** 2024-09-09

**Authors:** Marco Tramontano, Laura Casagrande Conti, Amaranta Soledad Orejel Bustos, Nicola Ferri, Tommaso Lelli, Ugo Nocentini, Maria Grazia Grasso, Andrea Turolla, Paolo Pillastrini, Leonardo Manzari

**Affiliations:** 1 Department of Biomedical and Neuromotor Sciences, University of Bologna, 40138 Bologna, Italy; nicola.ferri11@unibo.it (N.F.); andrea.turolla@unibo.it (A.T.); paolo.pillastrini@unibo.it (P.P.); 2 Unit of Occupational Medicine, IRCCS Azienda Ospedaliero-Universitaria di Bologna, 40138 Bologna, Italy; 3 Santa Lucia Foundation, Scientific Institute for Research and Health Care, 00179 Rome, Italy; laura.casagrandeconti@gmail.com (L.C.C.); a.orejel@hsantalucia.it (A.S.O.B.); tommi.lelli99@gmail.com (T.L.); u.nocentini@hsantalucia.it (U.N.); mg.grasso@hsantalucia.it (M.G.G.); 4 Department of Movement, Human and Health Sciences, University of Rome “Foro Italico”, 00135 Rome, Italy; 5 Department of Clinical Sciences and Translational Medicine, University of Rome Tor Vergata, 00133 Rome, Italy; 6 MSA ENT Academy Center, 03043 Cassino, Italy; lmanzari1962@gmail.com

**Keywords:** multiple sclerosis, vestibular, head impulse test, technology, rehabilitation, balance

## Abstract

Background: Multiple Sclerosis (MS) is the most prevalent autoimmune neurological condition in the world, leading to a wide variety of symptoms, including balance disorders. Objective: To evaluate the angular vestibulo–ocular reflex (aVOR) of all six semicircular canals (SCCs) through Head Impulse (HIMP) and Suppression HIMP (SHIMP) paradigms and any correlations with clinical balance scales. Methods: All participants were assessed using the Expanded Disability Status Scale (EDSS), Berg Balance Scale (BBS), and Mini-BESTest (MBT). Vestibular function was measured by video Head Impulse Test (vHIT), obtaining aVOR gain for each SSC. Results: Twenty-seven PwMS (mean age 47.93 ± 8.51 years old, 18 females) were recruited. Most of the patients (81.48%) presented abnormal aVOR gains for at least one SSC. A moderate to strong correlation between aVOR gains of the left anterior SSC and, respectively, the MBT and the BBS was found. The subgroup analysis, based on the EDSS class, confirmed the correlation with the BBS in the patients with the most significant disability. Conclusions: People with MS may present impairments of the aVOR in one or more semicircular canals. The aVOR gain impairment of the vertical semicircular canals correlates with balance and gait disorders identified through clinical scales in PwMS.

## 1. Introduction

Multiple Sclerosis (MS) is a chronic inflammatory, demyelinating, degenerative disease of the central nervous system that affects approximately 2.8 million worldwide [1,2]. People with MS (PwMS) present different symptoms depending on lesion location. The most common symptoms are fatigue and difficulty walking, which affect the activities of daily living and lead to disability. Difficulties in gait and balance disorders are the most common mobility limitations in PwMS [3]. Indeed, balance impairments are present in almost 86% of individuals with MS, and part of these disorders could be related to vestibular system dysfunction.

Rehabilitation plays a crucial role in improving functional outcomes, activity, and participation in PwMS, with moderate-quality evidence supporting its effectiveness even in the long term [4]. Specific training aimed to enhance the vestibular reflexes was found to be equally effective as other exercise-based interventions and more effective than no intervention in improving balance and dizziness symptoms in PwMS [5]. Some studies suggest a significant correlation between vestibular functioning and fatigue in PwMS [6], confirming its essential connections with balance and non-motor functions [7]. Although vestibular rehabilitation shows positive effects, one important limitation of these studies is the lack of an instrumental evaluation of the vestibular reflexes before and after the training [8,9]. 

In the last decades, new instrumental tools have been developed to assess vestibular function. A quick, well-tolerated, and safe vestibular test, the video Head Impulse Test (vHIT), was developed to measure the angular vestibulo–ocular reflex (aVOR) objectively [10]. In 2016, the vHIT was enhanced with the Suppressed Head Impulse (SHIMP) paradigm, a complementary variant of the traditional test that assesses the VOR gain [11]. Previous studies using the vHIT found that aVOR gain in the horizontal canals was significantly lower in PwMS with brainstem involvement compared to healthy people or PwMS without brainstem involvement, suggesting the use of vHIT to detect lesions in the brainstem vestibular pathway in PwMS [12,13]. Furthermore, worse aVOR gains and compensatory oculomotor functions were associated with a greater MS-related disability [14]. 

To the best of our knowledge, no study has been conducted to understand a correlation between aVOR alterations and balance disorders in PwMS, and no study compared the aVOR assessment through Head Impulse (HIMP) and SHIMP paradigms. Our hypothesis is that an altered function of the semicircular canals could be correlated to balance and gait impairment in PwMS. For this reason, the primary aim of this study is to evaluate the aVOR gains of all six semicircular canals through both HIMP and SHIMP paradigms. A secondary aim is to correlate the aVOR gain with the scores of the balance and gait scales.

## 2. Materials and Methods

This cross-sectional study was carried out at Santa Lucia Foundation Hospital and approved by the Local Independent Ethics Committee (Prot. CE/2022_011). All procedures contributing to this work comply with the ethical standards of the relevant national and institutional human experimentation guidelines and the World Medical Association Declaration of Helsinki. This article adheres to the Strengthening the Reporting of Observational Studies in Epidemiology (STROBE) guidelines [15]. 

### 2.1. Participants

Participants with a diagnosis of MS according to the McDonald criteria [16] were recruited and enrolled based on consecutive sampling at the Hospital between March 2022 and July 2023. The inclusion criteria were (i) diagnosis of relapsing–remitting (RRMS) or secondary-progressive (SPMS) MS diagnosed by a certified neurologist, (ii) over 18 years of age, (iii) Expanded Disability Status Scale (EDSS) [17] between 1 and 6, and (iv) ability to walk independently for at least 50 m. Exclusion criteria were (i) the presence of psychiatric and neurological disorders (other than MS) and other pathological conditions and/or clinical disorders severe enough to interfere with cognitive functioning and/or the performance of motor or cognitive tasks; (ii) the occurrence of clinical relapse in the three months prior to enrollment; (iii) steroid therapies administered in the 30 days preceding enrollment; (iv) the occurrence of a lower extremity fracture within three months prior to enrolment and/or other medical conditions that would interfere in the study procedures; and (v) history of vestibular disorders. All participants gave prior written consent.

### 2.2. Outcome Measures

At enrolment, clinical and demographic data were collected. A researcher not involved in the assessment performed a balance and gait assessment through the Berg Balance Scale (BBS) [18] and the Mini-BESTest scale (MBT) [19].

The BBS is a balance and fall risk assessment scale that includes 14 items; the total score ranges from 0 to 56, and the higher the score, the higher the function. 

The MBT is a 14-item scale designed to assess static balance and fall risk in adult populations; the total score ranges from 0 to 28, the latter indicating the highest level of function. It contains several sections, among which we considered ‘Walking’, with a minimum score of 0 and a maximum score of 10, with the same scoring direction as the MBT.

### 2.3. Instrumental Assessment of the VOR

The vHIT (ICS Impulse, Otometrics/Natus, Denmark) was used to evaluate the aVOR gain for movements in the direction and plane of the stimulation of the six semicircular canals, and the outcome was collected for both the HIMP and SHIMP paradigms. The assessment protocol followed strict and consistent procedures to reduce variability and obtain accurate and reliable data [20]. All the evaluations were performed by two physiotherapists specifically trained by an expert clinician (LM) and supervised during the study by a senior physiotherapist (MT) expert in the vestibular field. 

During the HIMP paradigm, the patient was instructed to fixate an earth-fixed point on the wall at a 1 m distance in front of them. Room lighting conditions were adjusted to ensure that the pupil was small and that its image was not affected by reflections at any point in the range of head movement. At each testing time, about 14 brief, rapid, horizontal head turns (head impulses) were applied to each side, always starting from the center, with unpredictable timing and direction with minimal bounce-back or overshoot at the end of the head impulse: each head impulse was “turn and stop”. The amplitude of the head rotation was about 10–15 deg, and the peak head velocity of the impulse was about 140–220 deg/s, with angular accelerations between about 3000 deg/s^2^ and 5000 deg/s^2^. Eye velocity and head velocity were recorded for each head turn. The usual measure of the adequacy of the vestibulo–ocular reflex in the HIMP paradigm is gain in terms of the quotient of eye velocity over head velocity at the peak of head acceleration; under physiological conditions, no compensatory saccades are expected [21,22]. To evaluate the vertical semicircular canals, the patient’s head was positioned approximately 35 degrees to the left for the RALP (Right Anterior–Left Posterior) plane test and 35 degrees to the right for the LARP (Left Anterior–Right Posterior) plane test. This positioning ensured the vertical canals were aligned with the head rotation plane, maximizing stimulation by head pitch movements. The clinician placed one hand on top of the patient’s head and the other under the chin. The head was then moved through a small angle (about 10–20 degrees) quickly and unpredictably. The intention was to move the skull rather than the skin to prevent the goggles from slipping relative to the eye, which would cause the appearance of an eye movement [23].

In the SHIMP paradigm, the patient is asked to follow a red dot on the wall generated by a laser attached to the top of the head while the clinician delivers head impulses; under physiological conditions, a saccade of opposite direction to the aVOR reflex after a short latency is expected. 

In the HIMP paradigm, we considered 0.76–1.29 a functional aVOR gain range for the vertical canals and 0.80–1.29 for the horizontal canals; we considered 0.66–1.29 a functional aVOR gain range in the SHIMP paradigm [21]. If the aVOR gain was outside these ranges, it was classified based on direction and defined as either a hypo-gain or hyper-gain [24].

### 2.4. Statistical Analysis

All the data were analyzed using SPSS software (IBM Corp. Released 2021. Version 28.0. Armonk, NY, USA: IBM Corp). Continuous data are presented as mean and standard deviation and categorical data are reported with frequencies (count and percentage). Spearman’s rank correlation tests were performed to assess the correlations between balance outcomes and VOR gains. There were no missing data. 

A sub-group analysis based on the severity of the EDSS score was performed considering Class 1 (EDSS = 1.0–3.5, n = 7) and Class 2 (EDSS = 4.0–6, n = 20).

## 3. Results

Twenty-seven PwMS (mean age 47.93 ± 8.51 years old, 18 females) were involved; clinical and demographic characteristics are reported in Table 1. The aVOR gain for 162 semicircular canals through the HIMP paradigm and 54 semicircular canals through the SHIMP paradigm was evaluated (Table 1). 

Approximately 81% of the enrolled participants exhibited abnormal aVOR gain in one or more semicircular canals when assessed using either the HIMP or SHIMP paradigm.

Specifically, 26.5% of the 162 canals in the HIMP paradigm and 18.5% of the 54 canals in the SHIMP paradigm were abnormal. In Table 2, the distributions of hyper-gain (i.e., VOR > 1.29) and hypo-gain (i.e., VOR < 0.76 for HIMP, VOR < 0.66 for SHIMP) were reported. 

The correlations between the VOR gain and clinical scale scores were analyzed as reported in Table 3. A significant correlation between the left anterior semicircular canal (HIMP) and the MBT and BBS scores was found. Instead, in the subgroup analysis based on the EDSS Classes, the BBS maintained a significant correlation with the left anterior semicircular canal in the patients of Class 2 (Table 4 and Table 5).

**Figure 1:**
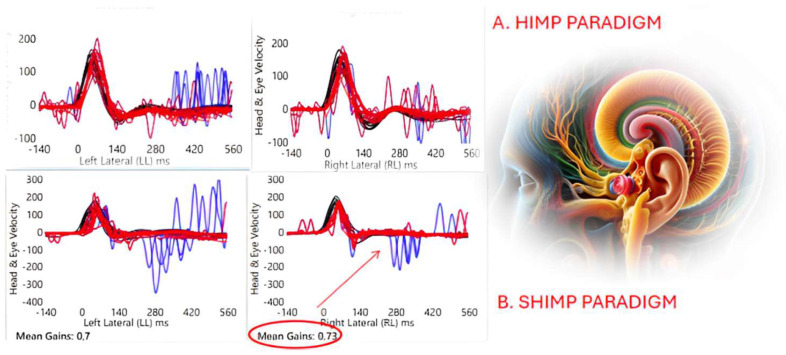
Absence of anti-compensatory saccades during the SHIMP paradigm.

The SHIMP paradigm assessment revealed that seven horizontal semicircular canals reported a functional aVOR gain and the absence of anti-compensatory saccades (Figure 1).

Horizontal semicircular canal function in both paradigms HIMP (A) and SHIMP (B) with video Head Impulse Test technology for one PwMS were objectively measured. Each panel shows a superimposed time series of head velocity (black for the head impulses) and the corresponding eye velocity (red) for the lateral canal dynamic function. The signs of head velocity for leftward impulses and eye velocity for rightward impulses have been inverted to allow for easier comparison. Functional horizontal angular vestibulo-ocular reflex (aVOR) gains are approximately 0.8–1.0 for the horizontal semicircular canals during the HIMP paradigm and 0.66–1.0 during the SHIMP. In the SHIMP paradigm (B) for rotations toward the right side, aVOR gain is 0.73. The red arrow indicates the unexpected absence of the aVOR suppression strategies (anti-compensatory saccades).

## 4. Discussion

This study aimed to assess vestibular semicircular canal function in PwMS using vHIT to ascertain the angular vestibular reflex related to the stimulation of all six semicircular canals. Our findings revealed that PwMS may have aVOR dysfunction, with a significant correlation between the aVOR gains of the vertical canals and balance clinical scales. From a rehabilitative standpoint, these correlations could be significant and may guide the decision-making process when customizing rehabilitative programs to improve dynamic postural and gait stability. Indeed, training the aVOR reflex could potentially enhance balance and gait in PwMS [9,21,25,26,27,28,29,30]. Future studies should introduce instrumental vestibular assessment as an outcome measure to evaluate the enhancement of the vestibular reflexes after rehabilitation.

People with distinct levels of disability were involved, and we found a different correlation between aVOR gains and clinical scale scores if analyzed separately according to the EDSS classification. These results are consistent with the positive correlation found by Grove and colleagues [14] between the severity of disability in PwMS and an altered vestibular function. Furthermore, the significant correlation of the BBS with vestibular function in EDSS Class 2 alone is consistent with the role of the EDSS cutoff in identifying individuals with abnormalities in balance system integration [31]. 

Our study revealed isolated canal dysfunctions in PwMS, which differ from those typically observed in the various stages of vestibular unilateral hypofunction [32]. These isolated dysfunctions may be attributed to demyelination plaques manifesting within the vestibular pathways, leading to dizziness and balance disorders [33]. Specifically, these plaques could develop in the brainstem, where both efferent and afferent vestibular pathways coexist, contributing to the observed vestibular impairments. Additionally, inflammatory demyelination has been frequently identified in the vestibular nuclei and the root entry zone of the eighth cranial nerve, further supporting the connection between central nervous system lesions and vestibular dysfunction in PwMS.

Demyelination in peripheral neural connections within the inner ear structures may also play a role in these isolated dysfunctions, potentially exacerbating balance and gait disorders in PwMS. Our analysis indicated a qualitative reduction in aVOR gains across multiple semicircular canals, suggesting a potential generalized impairment in vestibular function in this population. 

The SHIMP paradigm provided valuable insights into the central nervous system’s ability to suppress aVOR through visual input, known as visuo–vestibular interaction. However, it is important to note that the SHIMP paradigm primarily assesses aVOR suppression in the plane of the horizontal semicircular canals. Therefore, potential alterations in the suppression mechanism in the vertical semicircular canals remain unassessed with the current methodology. This limitation highlights the need for further research to determine whether the observed reductions in slow-phase velocity in the vertical canals are due to peripheral or central lesions. Interestingly, some PwMS exhibited functional aVOR gains without anti-compensatory saccades in the SHIMP test, which may indicate a lack of central suppression of the aVOR due to lesions in the central pathways. This finding suggests that central regulatory mechanisms of the aVOR, processed in the vestibular nuclei complex within the brainstem, may be compromised in PwMS.

In our sample, we found a significative prevalence of abnormal aVOR gains in PwMS, with both hypo- and hyperfunctions. This finding aligns with our previous study focused on stroke survivors in the sub-acute and chronic phases where impairment of the semicircular canal was observed [34]. While hypofunctions are more easily interpreted from a clinical point of view, hyperfunctions have not yet been fully framed in scientific literature to date. Choi and colleagues [35] observed increased aVOR gains in diffuse cerebellar lesions, followed by a corrective backup saccade in the opposite direction than the overt saccade typically seen during vHIT. These authors hypothesized that this could suggest initial cerebellar involvement. One possible explanation for this phenomenon is the reduced inhibitory control exerted by the cerebellum on vestibular reflexes [36]. This mechanism is akin to what occurs in cerebellar ataxia, where aVOR gain is typically decreased in the horizontal canals but increased in the vertical canals [37]. The processing of vestibular semicircular canal function primarily takes place in the brainstem, within the vestibular nuclei complex. Information from the semicircular canals is relayed by the first neuron of the reflex arc to the superior and medial vestibular nuclei. Second-order neurons then relay impulses through the medial longitudinal fasciculus to the III, IV, and VI cranial nerves via ipsilateral and contralateral connections. Finally, third-order neurons send excitatory and inhibitory impulses to the corresponding extraocular muscles, generating eye movements equal in speed and amplitude but opposite to head movements, thereby stabilizing the image on the fovea.

Given the complex integration of afferent signals across multiple structures, it is challenging to attribute the observed aVOR impairments solely to peripheral lesions. The possibility of central lesions in the reflex arc due to demyelination in the pons or other brainstem areas cannot be ruled out. This complexity underscores the necessity of a comprehensive approach to understanding and managing vestibular dysfunction in PwMS.

The impairments in aVOR gain observed in PwMS contribute to a deeper understanding of vestibular function in this population, addressing a significant gap in the current literature. Future research should explore the underlying mechanisms of semicircular canal dysfunction in PwMS more thoroughly, particularly through longitudinal studies that could elucidate the natural history of aVOR gain impairments and their impact on long-term outcomes. 

The diagnosis and treatment of vestibular diseases can vary due to differences in assessment tools and outcome measures used by healthcare providers. Standardization efforts and an interdisciplinary approach can help improve the consistency and effectiveness of care for individuals with balance disorders. Additionally, innovative rehabilitation strategies, including tailored vestibular physical therapy exercises, could be developed to optimize balance, mobility, and overall quality of life for individuals living with MS and aVOR dysfunction. Multiple sensory information is involved in balance control, and proprioceptive, visual, and vestibular cues are integrated by the central nervous system depending on the environmental conditions, according to the motor activities the person engages in their daily activities [38,39,40].

Our study has some limitations. The observational design implies not being able to infer a causal link between MS and abnormal aVOR gain; we cannot attribute aVOR anomalies with certainty to the underlying pathology, to the reduction in physical activity resulting from disability, or to other factors not considered. Additional vestibular testing, such as rotational chair testing and caloric vestibular stimulation, was not performed. Moreover, the sample size was small, and 74% of the participants included exhibited a moderate level of disability. This could potentially compromise the robustness and generalizability of our findings. Finally, we did not compare the vHIT with the clinical Head Impulse Test (HIT), which is usually performed as a bedside test; this, in relation to recent literature [35], could have confirmed the discrepancy between clinical and video-assisted HIT, with patterns related to central lesions. Furthermore, future research will aim to address the limitations identified in this study, including the expansion of the patient cohort and the investigation of VOR impairments in PwMS with comorbid vestibular conditions, such as Vestibular Migraine. Additionally, incorporating a wider range of vestibular tests will help to further elucidate the complex vestibular dysfunctions in PwMS.

## 5. Conclusions

PwMS may present impairments of the aVOR in one or more semicircular canals. The aVOR gain impairment of the vertical semicircular canals correlates with balance and gait disorders identified through clinical scales. Both HIMP and SHIMP paradigms could be valid strategies to assess vestibular canal function in PwMS. Further studies should confirm our results to consolidate the use of the instrumental VOR assessment in the clinical management of the PwMS.

## Figures and Tables

**Table 1:** Characteristics of participants.

Sex F (%)	66.67	
Age (years)	47.93 ± 8.51	
Stature (m)	1.70 ± 0.09	
Weight (kg)	66.4 ± 15.05	
EDSS 1.0–3.5 (%)	25.93	
EDSS 4.0–6.5 (%)	74.07	
Time from diagnosis (years)	16.22 ± 10.02	
Education (years)	13.89 ± 2.98	
Mini-BESTest	17.30 ± 5.74	
Walking MBT	6.00 ± 2.13	
BBS	44.67 ± 8.85	
HIMP aVOR gain (mean ±SD)	Left	Right
Anterior	0.78 (±0.22)	0.86 (±0.14)
Horizontal	0.92 (±0.19)	0.98 (±0.24)
Posterior	0.82 (±0.12)	0.76 (±0.17)
SHIMP aVOR gain (mean ±SD)	Left	Right
Horizontal	0.78 (±0.21)	0.87 (±0.23)

EDSS: Expanded Disability Status Scale; MBT: MiniBESTest; BBS: Berg Balance Scale; Walking MBT: refers to the Walking section of MBT; ± = standard deviation.

**Table 2:** Percentages of abnormal VOR gain for both HIMP and SHIMP paradigms.

		Abnormal (n)	Hypo- or Hyper-Gains (%)	Total Abnormal Gains (%)
HIMP VOR Gain				
Right Horizontal	<0.76	2	7.41	8.52
	>1.29	3	1.11	
Left Horizontal	<0.76	2	7.41	11.11
	>1.29	1	3.70	
Left Anterior	<0.76	9	33.33	37.03
	>1.29	1	3.70	
Right Posterior	<0.76	14	51.85	51.85
	>1.29	0	0	
Right Anterior	<0.76	4	14.81	14.81
	>1.29	0	0	
Left Posterior	<0.76	7	25.93	25.93
	>1.29	0	0	
SHIMP VOR gain				
Right Horizontal	<0.66	4	14.81	18.51
	>1.29	1	3.70	
Left Horizontal	<0.66	5	18.52	18.52
	>1.29	0	0	

HIMP: Head Impulse Paradigm; SHIMP: Suppression Head Impulse Paradigm.

**Table 3:** Correlation between VOR gain and clinical scale scores.

N = 162 Semicircular Canals	Walking MBT	MBT	BBS
HIMP aVOR Gain			
Right Horizontal	−0.005	0.068	0.069
Left Horizontal	−0.092	−0.072	−0.055
Left Anterior	0.203	0.428 *	0.465 *
Right Posterior	0.212	0.191	0.163
Right Anterior	0.014	0.122	0.129
Left Posterior	0.101	0.142	0.143
**N = 54 semicircular canals** **SHIMP aVOR gain (mean ± SD)**			
Right Horizontal	0.153	0.014	−0.069
Left Horizontal	−0.165	−0.172	−0.295

* Correlation is significant at the 0.05 level (two-tailed). MBT: MiniBESTest; BBS: Berg Balance Scale; Walking MBT: refers to the Walking section of MBT; HIMP: Head Impulse Paradigm; SHIMP: Suppression Head Impulse Paradigm.

**Table 4:** Correlations in Class 1 (EDSS 1.0–3.5).

N = 42 Semicircular Canals	Walking MBT	MBT	BBS
HIMP aVOR Gain			
Right Horizontal	0.200	0.252	0.200
Left Horizontal	−0.127	−0.072	−0.273
Left Anterior	0.018	0.018	0.202
Right Posterior	0.431	0.391	0.266
Right Anterior	−0.257	−0.209	−0.055
Left Posterior	0.385	0.400	0.385
**N = 14 semicircular canals** **SHIMP aVOR gain**			
Right Horizontal	0.436	0.414	0.255
Left Horizontal	0.436	0.396	0.073

MBT: MiniBESTest; BBS: Berg Balance Scale; Walking MBT: refers to the Walking section of MBT; HIMP: Head Impulse Paradigm; SHIMP: Suppression Head Impulse Paradigm; EDSS: Expanded Disability Status Scale.

**Table 5:** Correlations in Class 2 (EDSS 4.0–6.5).

N = 120 Semicircular Canals	Walking MBT	MBT	BBS
		
HIMP aVOR Gain			
Right Horizontal	−0.163	−0.077	−0.051
Left Horizontal	−0.176	−0.169	−0.107
Left Anterior	0.105	0.398	0.491 *
Right Posterior	0.177	0.207	0.178
Right Anterior	0.022	0.135	0.135
Left Posterior	0.096	0.103	0.142
**N = 40 semicircular canals** **SHIMP aVOR gain**			
Right Horizontal	0.192	0.009	−0.042
Left Horizontal	−0.181	−0.135	−0.196

* Correlation is significant at the 0.05 level (two-tailed). MBT: MiniBESTest; BBS: Berg Balance Scale; Walking MBT refers to the Walking section of MBT; HIMP: Head Impulse Paradigm; SHIMP: Suppression Head Impulse Paradigm; EDSS: Expanded Disability Status Scale.

## Data Availability

Data are available upon reasonable request to the corresponding author.
